# Does burnout among doctors affect their involvement in patients' mental health problems? A study of videotaped consultations

**DOI:** 10.1186/1471-2296-10-60

**Published:** 2009-08-26

**Authors:** Else M Zantinge, Peter FM Verhaak, Dinny H de Bakker, Klaas van der Meer, Jozien M Bensing

**Affiliations:** 1 NIVEL, Netherlands Institute for Health Services Research, Utrecht, the Netherlands; 2 Current: National Institute for Public Health and the Environment (RIVM), Centre for Public Health Forecasting, Bilthoven, the Netherlands; 3 Department of General Practice, University of Groningen, Groningen, the Netherlands

## Abstract

**Background:**

General practitioners' (GPs') feelings of burnout or dissatisfaction may affect their patient care negatively, but it is unknown if these negative feelings also affect their mental health care. GPs' available time, together with specific communication tools, are important conditions for providing mental health care. We investigated if GPs who feel burnt out or dissatisfied with the time available for their patients, are less inclined to encourage their patients to disclose their distress, and have shorter consultations, in order to gain time and energy. This may result in less psychological evaluations of patients' complaints.

**Methods:**

We used 1890 videotaped consultations from a nationally representative sample of 126 Dutch GPs to analyse GPs' communication and the duration of their consultations. Burnout was subdivided into emotional exhaustion, depersonalisation and reduced accomplishment. Multilevel regression analyses were used to investigate which subgroups of GPs differed significantly.

**Results:**

GPs with feelings of exhaustion or dissatisfaction with the available time have longer consultations compared to GPs without these feelings. Exhausted GPs, and GPs with feelings of depersonalisation, talk more about psychological or social topics in their consultations. GPs with feelings of reduced accomplishment are an exception: they communicate less affectively, are less patient-centred and have less eye contact with their patients compared to GPs without reduced accomplishment.

We found no relationship between GPs' feelings of burnout or dissatisfaction with the available time and their psychological evaluations of patients' problems.

**Conclusion:**

GPs' feelings of burnout or dissatisfaction with the time available for their patients do not obstruct their diagnosis and awareness of patients' psychological problems. On the contrary, GPs with high levels of exhaustion or depersonalisation, and GPs who are dissatisfied with the available time, sometimes provide more opportunities to discuss mental health problems. This increases the chance that appropriate care will be found for patients with mental health problems. On the other hand, these GPs are themselves more likely to retire, or risk burnout, because of their dissatisfaction. Therefore these GPs may benefit from training or personal coaching to decrease the chance that the process of burnout will get out of hand.

## Background

Feelings of job dissatisfaction and job stress are problems shared by general practitioners (GPs) in many countries [1-5]. GPs report a lack of time and heavy workload as the main causes for these feelings of discontent and stress [6-9]. These negative feelings may in the long-term lead to burnout [10,11].

Burnout is 'a syndrome of emotional exhaustion, depersonalisation, and reduced personal accomplishment that can occur among individuals who work with people in some capacity' [12]. Emotional exhaustion is the key aspect of burnout, and refers to feelings of energy depletion. Emotional exhaustion can initiate the burnout syndrome: exhaustion may evoke depersonalisation and feelings of reduced accomplishment [13]. Depersonalisation is expressed in a negative, cynical and distant attitude towards others. Reduced personal accomplishment is a negative attitude to oneself, in relation to one's job.

GPs' dissatisfaction and burnout do not only affect the GP's own well-being, but it may also have consequences for health policy and for patient care. Job dissatisfaction is a major cause of GP turnover [3,9] and it may add to a negative image of the profession. This can lead to shortages of GPs, a main concern for health policy makers. Moreover, doctors' feelings of discontent can damage the quality of patient care [14,15]. Other studies showed that exhaustion and burnout are associated with more self-reported medical errors [15,16], although another study demonstrated that burnout is not associated with an objective measure of medical errors [17].

One of the perspectives to explain burnout is found in equity theory [18,19]. This theory is already applied to general practitioners in earlier studies [10,13].

According to equity theory, people evaluate their relationships with others in terms of input (investments, job demands) and output (outcomes, rewards), compared to others around them. This principle can also be applied to work situations. In work settings people compare their job demands and their investments with the rewards they receive. When job demands are high, or rewards are low, people may experience an inequity or imbalance. But 'equity is in the eye of the beholder' [19]: the evaluation of the balance between job demands and rewards is dependent on personal factors.

According to equity theory, people who experience an imbalance are strongly motivated towards restoring this imbalance. People who experience an imbalance between their investments and rewards develop feelings of distress, and a long-lasting period of stress may eventually lead to burnout [10,11]. Solutions to correct the imbalance can be found in decreasing the job demands, adjusting one's expectations, or increasing the rewards. Decreasing the job demands is the most obvious solution, as it is demonstrated that high job demands are more strongly related to burnout than a lack of rewards [20].

Although little specific information is available about how GPs' negative feelings are reflected in their patient interactions, it is possible to imagine what happens when a GP is troubled with feelings of burnout or dissatisfaction. According to the perspective of equity theory, GPs with high levels of burnout experience an imbalance between their job demands and rewards, and will try to restore this imbalance. One can hypothesise that GPs who are exhausted and cynical toward their patients, and suffer from feelings of worthlessness, will invest less in their patient contacts than other GPs. Their distant, cynical attitude that characterises burnout, will, in particular, reduce their openness and affect their attitude towards their patients. Also, GPs who are dissatisfied with their job and their available time are expected to invest less in their patients, and to shorten their consultations, in order to gain time and energy to restore the imbalance.

One of the aspects of a GP's job that demands extra time and energy, according to GPs themselves, are their patients' mental health problems [21,22]. The GP has an important position in this, as they are often the first health professionals to be contacted by patients with mental health problems [23]. GPs as generalists are the people assigned to provide integrated care for both patients' somatic and psychological problems. Early identification of patients' mental health problems is important, because it is the first step in finding adequate care for the patient.

Time is an important condition for discussing psychological problems in the consultation. It is known that consultations that include psychological problems take more time [24,25], and doctors experience more frequently a lack of time [25]. GPs mentioned this lack of time as an obstacle to detecting and treating patients with psychological problems in the consultation [26,27]. Also, patients themselves mention lack of time as one of the reasons for not presenting psychological problems in the consultation [28]. But time alone is not enough to provide adequate psychological care. Furthermore, specific communication tools are required to stimulate the patient to disclose their psychological problems [29,30]. Aspects of GPs' communication that are associated with an increase of psychological aspects in the consultation are GPs' affective behaviour, that is being patient-centred [31,32], asking questions about psychological or social issues [29,32] and showing eye contact with their patients [33,34].

Given the importance both of time and specific communication tools for discussing mental health problems, and given our presumption that burnout and dissatisfaction in particular affect GPs' available time and their affective approach in patient interactions, we expect the following:

GPs with burnout or dissatisfaction with the available time will, in order to restore the balance and gain time and energy, be less inclined to get involved in their patients' mental health problems, compared to GPs with low levels of burnout, or GPs who are satisfied with the available time. We expect that GPs with high burnout levels, or dissatisfaction with the available time, will adapt their communication to elicit less patient disclosure with respect to their mental health problems. There will be less encouragement for their patients to discuss their mental health problems, resulting in less involvement of psychological aspects in the consultation.

Therefore, we investigate in this paper:

Do GPs with high levels of burnout, and GPs who are dissatisfied with the available time

• Have shorter consultations?

• Show less affective communication in their consultations?

• Talk less frequently about psychosocial issues?

• Make less psychological evaluations?

## Methods

### Design

Secondary analyses were performed on data from the second Dutch National Survey of General Practice (DNSGP-2), a cross-sectional study conducted in the Netherlands in 2000–2002 [35]. 195 GPs in 104 general practices participated in this National Survey. Data are derived from several sources within the National Survey: 1) a video registration of consultations, 2) a short questionnaire that GPs completed after each consultation about the patient and the consultation and 3) a written GP questionnaire about a wide range of topics.

The study was carried out according to Dutch legislation on privacy [35]. The privacy regulation of the study was approved by the Dutch Data Protection Authority. According to Dutch legislation, approval by a medical ethics committee was not obligatory for this observational study.

### Subjects

142 of the 195 GPs gave permission to videotape consultations during one or more days. This sample of 142 GPs is representative of the Dutch population of GPs with regard to their age, sex, education, length of residence, degree of urbanisation and number of working hours [36].

88.1% of the visiting patients gave written informed consent to participate in the study. Approximately 15 consultations per GP were observed by trained observers.

126 of the 142 GPs who participated in the video registration completed a questionnaire, including questions about burnout and job satisfaction. These 126 GPs are representative in their levels of burnout and job satisfaction compared to all GPs from the National Survey that completed the GP questionnaire (n = 164). In total 1890 consultations from 126 GPs were suitable for analyses in this paper.

### Measures

#### Burnout (independent)

GPs' levels of burnout were measured using the UBOS, Utrecht Burnout Scale [37], a Dutch version of the Maslach Burnout Inventory [12]. We made use of the UBOS-C, a variant of the UBOS that has been developed for providers of human services, according to the MBI-Human Services Survey. The UBOS-C provides reliable measures of aspects of burnout, comparable to the reliability of the MBI [37]. The UBOS-C consists of 20 items, eight referring to feelings of emotional exhaustion, five to depersonalisation and seven to personal accomplishment. The scores range from 0 = never to 6 = always. Mean scores for these three components of burnout are calculated for each GP, taking into account the maximum allowed number of missing items [12]. For each subscale, GPs were classified in three groups, referring to low, middle and high scores on the subscale. Cut off points for low, middle and high burnout scores were derived from the group norms for Dutch primary care providers (n = 1523), as published in the manual of the UBOS [37]. Cut off points for low and high levels of burnout are presented in table 1.

**Table 1:** Cut off point for low and high levels of the subscales of burnout

	**Low**	**High**
Emotional exhaustion	< = 1.24	> = 2.75
Depersonalisation men	< = 0.79	> = 2.20
Depersonalisation women	< = 0.59	> = 1.60
Personal accomplishment	< = 3.70	> = 4.71

#### Job satisfaction (independent)

GPs completed a job satisfaction scale in the GP questionnaire, originally derived from McCranie (1982) [38]. According to a list of 16 working activities, the GPs recorded their satisfaction with that specific aspect of their job on a 5-point scale, ranging from 1 = very dissatisfied, 2 = dissatisfied, 3 = partly satisfied and partly unsatisfied, 4 = satisfied, to 5 = very satisfied. A mean score on this scale was calculated to measure general job satisfaction, with a Cronbach's alpha of .83 (1 item deleted). The job satisfaction questionnaire was subdivided into three factors, referring to different aspects of job satisfaction [39]. We made use of the subgroup 'satisfaction with the available time', with a Cronbach's alpha of .74. Three categories were constructed for the 'general job satisfaction' scale and for the 'satisfaction with the available time' scale: GPs with mean scores of 1 till 2.5 are indicated as dissatisfied; GPs scoring 2.5–3.5 are classified as moderately satisfied, and GPs with a mean scores of 3.5 and above are indicated as satisfied.

#### GPs' communication (dependent)

The videotaped consultations were rated by trained observers for several aspects of GPs' communication. Verbal communication was rated according to the Roter Interaction Analysis System (RIAS), a widely used and validated observation instrument for coding verbal communication in medical interactions [40,41]. The system is developed to code both doctor and patient communication. The unit of analysis is the smallest meaningful string of words, called 'utterance'. The version of the RIAS that is used for the DNSGP-2, distinguishes 8 categories of affective or social-emotional behaviour and 18 categories of instrumental or task-oriented behaviour. All categories are mutually exclusive. From the cluster 'affective communication', we selected GPs' utterances with respect to empathy, showing partnership and support and legitimising (further called: 'empathy'), and secondly, GPs' concern shown toward their patients.

From the group 'instrumental communication' the following utterances are selected: 1) biomedical talk, referring to questions, information and counselling about biomedical subjects and, 2) psychosocial talk, referring to questions, information and counselling about psychological or social topics.

Other aspects of communication that are studied are GPs' patient-centeredness and the percentage of eye contact in the consultation. GPs' patient-centeredness was determined by observers by rating three dimensions of patient-centeredness, coded from 1 (not at all) to 5 (to a great extent). These dimensions are: giving room to the patient, shared decision-making, and showing openness [36]. Ratings on each of these dimensions are integrated in an average patient-centeredness scale ranging from 1 to 5, with Cronbach's alpha .74. Inter-rater reliability for GP communication, expressed in Pearson's correlation coefficients, varied between .72 and .95 [36].

The GP's eye contact is indicated as the percentage of total consultation time the GP has eye contact with the patient.

#### The GP's psychological evaluations and length of consultation

The GPs registered after each consultation whether psychological aspects play a part in the patient's complaints. They recorded this on a five point scale ranging from 1 = 'psychological aspects play no part at all', to 5 = 'psychological background'. This is interpreted as the GP's 'psychological evaluation'.

In each consultation, one or more diagnoses of the patients were coded by observers, according to the International Classification of Primary Care (ICPC) [42]. A distinction was made between consultations with one or more diagnoses in ICPC chapter P 'Psychological' or Z 'Social', and consultations with only somatic diagnoses.

Afterwards, observers measured consultation length in minutes to two decimal places. Interruptions, such as telephone calls, were subtracted from the total consultation time.

### Analyses

Analyses were performed on the levels of the GP and the consultation. On the GP level, descriptive statistics, Pearson's correlation coefficients and Cronbach's alphas, were calculated for the components of burnout and job satisfaction, making use of SPSS 11.5 software.

On the consultation level, multilevel regression analyses were performed, using MlWin 2.0 software. Multilevel analyses were necessary due to the two-level structure of the data with level 1 being the consultation, and level 2, the GP.

First, multilevel regression analyses were performed with, respectively, the GP's patient-centeredness, the percentage of eye contact, the length of consultation and the GP's psychological evaluation, as dependent measures, using a normal distribution model. Predictors in all models were the GP's level of burnout and job satisfaction (low versus high), and the GP's and patient's sex and age were included as potential confounders. Mean scores of the outcome measures were calculated. We adjusted for clustering at the GP level by using a random intercept.

Second, the GP's communication utterances were analysed using a Poisson distribution model, with extra Poisson variation to account for over-dispersion. The Poisson models were fitted using a second order Penalized Quasi-Likelihood estimation.

Finally, the presence or not of a psychological or social diagnosis was analysed using a binomial logit model.

## Results

In table 2, descriptive statistics are presented for GPs' levels of burnout, general job satisfaction, and satisfaction with the available time. A **higher **score on emotional exhaustion and depersonalisation means that GPs have more feelings of exhaustion or depersonalisation. A **lower **level of personal accomplishment or satisfaction corresponds with reduced accomplishment or dissatisfaction.

**Table 2:** Descriptive statistics for GPs' levels of burnout (range 0–6) and job satisfaction (range 1–5)

	All 126 GPs	GPs with high levels of burnout/dissatisfaction	
		
	Mean (sd)	N (%)	Mean (sd)
Emotional exhaustion	1.58 (.79)	9 (7%)	3.33 (1.66)
Depersonalisation	1.32 (.72)	14 (11%)	2.67 (.64)
Personal accomplishment	4.27 (.77)	28 (22%)	3.23 (.35)
			
General job satisfaction	3.25 (.45)	6 (5%)	2.28 (.15)
Job satisfaction time	2.97 (.61)	33 (26%)	2.18 (.23)

Sd = standard deviation

Mean scores in table 2 show that GPs' feelings of emotional exhaustion, depersonalisation and reduced accomplishment are, on average, found 'seldom' or 'sometimes', according to the meaning of the scale points. Table 2 shows that 7% of the GPs reported high levels of exhaustion, 22% showed high levels of reduced personal accomplishment and 11% scored high on the depersonalisation scale.

Table 2 also shows that 5% of the GPs are not satisfied with their job. The number of GPs that are not satisfied with the available time is more than a quarter. GPs are especially dissatisfied with their leisure time and the time they have to manage their practice (not in table). From all the items referring to satisfaction with the available time, GPs are most satisfied with the available patient time.

Because there was only a small subgroup of six GPs with a low general job satisfaction, we answer the research questions by focusing this article on the GP's satisfaction with the available time.

Table 3 shows sex and age distributions of GPs with low and high levels of burnout and job satisfaction with the available time. Female GPs more often have feelings of exhaustion and depersonalisation, but their feelings of accomplishment are higher, compared to men. Male GPs are more often dissatisfied with the available time compared to female GPs. Age differences between GPs with low and high levels of burnout and job satisfaction with the available time are not substantial.

**Table 3:** Correlations and Cronbach's alphas (diagonally) for burnout subscales and dissatisfaction with the available time (n = 126)

	EE	DP	PA	JS time
Emotional exhaustion (EE)	(.88)	-	-	-
Depersonalisation (DP)	.56**	(.76)	-	-
Personal accomplishment (PA)	-.21*	-.32**	(.81)	-
Job satisfaction (JS) time	-.45**	-.20*	.12	(.74)

* p < .05; ** p < .01

In table 4, Pearson's correlations between the burnout subscales and job satisfaction with the available time are presented. On the diagonals, the internal consistency of each subscale, expressed in Cronbach's alphas, is shown.

**Table 4:** Gender and age of 126 GPs with low, middle and high levels of burnout and satisfaction with the available time

	N (%)	% male GPs	Average age GPs
EE low	54 (43%)	74%	47
EE high	9 (7%)	56%	44
DEP low	31 (25%)	77%	47
DEP high	14 (11%)	57%	45
PA low	28 (22%)	82%	46
PA high	41 (33%)	76%	47
JS time low	33 (26%)	85%	46
JS time high	27 (21%)	70%	49

EE = Emotional Exhaustion; DEP = Depersonalisation; PA = Personal accomplishment; JS = job satisfaction.

GPs who are more exhausted more often also have feelings of depersonalisation and reduced accomplishment. Additionally, exhausted GPs are less satisfied with the available time. GPs with higher depersonalisation levels more often have feelings of reduced accomplishment and are more often dissatisfied with the available time. No significant correlations were found between personal accomplishment and job satisfaction with the available time. Cronbach's alphas show a satisfactory internal consistency in the scales of burnout and satisfaction.

In table 5, GPs with low and high levels of burnout and satisfaction with the available time, are compared, by means of multilevel analyses, with respect to their communication, eye contact, patient-centeredness, length of consultation and awareness of psychological problems. Estimated means presented in table 5 are corrected for the age and gender of the GPs and patients.

**Table 5:** Adjusted^a ^means for communication utterances and communication aspects, and outcomes of the consultations (N = 131–795)

	**Emotional exhaustion**		**Depersonalisation**		**Pers. accomplishment**		**Satisfaction time**	
	Low	High	Low	High	Low	High	Low	High
	N = 786–795	N = 131–132	N = 454–460	N = 207–208	N = 406–411	N = 600–607	N = 476–484	N = 396–400

**Instrumental utterances**	65.2	82.6**	69.1	74.9	64.9	73.6*	75.1	61.8**
from which:								
- biomedical talk	39.9	47.5*	42.7	42.9	38.2	45.2**	45.5	38.1**
- psychosocial talk:	6.6	12.0**	6.1	9.7**	7.0	7.7	7.8	6.2
* psychosocial questions	2.9	4.2**	2.7	3.9**	3.1	3.1	2.8	2.7
* psychosocial information	2.9	6.2**	3.2	4.8**	4.0	3.4	4.3	3.2
* psychosocial counselling	.6	1.2**	.6	.8	.6	.7	.7	.6
								

**Affective utterances**	44.4	47.9	47.0	47.8	42.3	49.8*	47.9	41.3
from which:								
- empathy	1.3	1.6	1.3	1.5	1.4	1.5	1.4	1.1
- showing concern	.2	.2	.2	.3	.2	.2	.1	.2

**Other communication**								
Patient-centeredness	3.8	3.7	3.8	3.8	3.8	3.9**	3.8	3.8
% eye contact	42%	42%	43%	39%	40%	44%*	39%	41%

**Outcomes**								
Consultation length	9.3	11.4**	9.8	10.7	9.4	10.2	10.7	8.9**
Psychological evaluation	2.5	2.6	2.7	2.7	2.5	2.6	2.6	2.5
Psychol/social diagnosis	9%	12%	9%	11%	9%	10%	9%	9%

^a ^Adjusted for the GPs' and patients' age and gender

* p < .05; ** p < .01

Table 5 shows that exhausted and dissatisfied GPs have consultations that are roughly two minutes longer compared to GPs who are not exhausted or dissatisfied. The longest consultations are consultations from GPs who reported feelings of exhaustion – on average 11.4 minutes. GPs who are satisfied with the available time have the shortest consultations: 8.9 minutes on average. GPs with feelings of exhaustion or dissatisfaction and GPs who feel competent, show more instrumental communication in their consultations. Exhausted GPs and GPs with feelings of depersonalisation talk more frequently about psychological or social issues than GPs without feelings of exhaustion or depersonalisation. They ask more questions about psychological or social topics, give more information, and exhausted GPs show also more psychosocial counselling in their consultations. The extra instrumental communication that dissatisfied GPs and GPs who feel competent show in their consultations is due to their biomedical utterances. No differences in psychosocial communication are found.

The number of a GP's affective utterances is only significantly higher in consultations from GPs who feel competent compared to GPs with feelings of low accomplishment. GPs who feel competent are also more patient-centred in their consultations and show more eye contact.

Table 5 shows that the GP's level of burnout or satisfaction with the available time, is not associated with differences in their awareness of patients' psychological problems. GPs do not make more psychological evaluations or diagnoses in their consultations when they have feelings of burnout or are dissatisfied with their available time.

## Discussion

### Main findings

Contrary to the expectations, GPs with feelings of burnout or dissatisfaction do not have shorter consultations. Also the other expectations, suggesting that GPs with high levels of burnout or dissatisfaction would show less affective communication, talk less frequently about psychological and social issues and are less aware of psychological problems in their patients, are not confirmed.

On the contrary, our findings showed that exhausted GPs and GPs who are dissatisfied with the available time have longer consultations, and they show more communication in total in their consultations. This extra communication is expressed in more talking about psychological or social issues by exhausted GPs, while dissatisfied GPs speak in more biomedical terms. GPs with high levels of depersonalisation also speak in more psychosocial terms with their patients, compared to GPs with low depersonalisation.

But although the GPs' higher levels of burnout or dissatisfaction are associated with more communication and longer consultations, this is not translated into more psychological evaluations of their patients' complaints.

One exception to the main conclusion is the result showing GPs who feel incompetent, with low scores on 'personal accomplishment'. They communicate in their consultations for the greater part as we expected: They show less affective communication, are less patient-centred and have less eye contact with their patients, compared to GPs who feel competent.

### Discussion of results

How can we explain these unexpected findings? There are basically two perspectives to discuss our results. The first was the perspective that was the focus of this paper: the idea that GPs' negative feelings would be reflected in the consultation. In line with this perspective, several alternative explanations for our findings can be discussed.

Firstly, this study showed that GPs with high exhaustion and depersonalisation levels, talked more frequently about psychological or social issues with their patients. Maybe GPs who have feelings of distress themselves are more focused on psychological aspects in their patients because of their affinity with those kinds of problems.

An alternative explanation for the finding that consultations from GPs with feelings of burnout take longer is that these GPs are less effective in their consultations.

Another notable result is that GPs who are dissatisfied with the available time have longer consultations containing more communication. It is known from earlier studies that GPs who are dissatisfied with their jobs are especially dissatisfied with the organisation and paperwork that their job brings with it [1,3,7], while their patient care even contributes to their job satisfaction. According to equity theory, it is possible that dissatisfied GPs in our study will focus more on their patient contacts, while at the same time trying to limit their involvement in other aspects of their job in order to regain some balance.

Finally, results in this study show that GPs' awareness of psychological problems in their patients is not dependent of their feelings of burnout or dissatisfaction. These results correspond with results from our previous study, in which it was demonstrated that the presence and severity of mental distress in the patient are more important reasons for a GP to take psychological aspects into consideration than their workload [43]. And, of course, the reality is complex. A lot of other influences play a part as determinants of GPs' consulting style, such as the personality of a GP.

A second perspective from which to discuss the results is that maybe the nature of GPs' patient contacts determines GPs' negative feelings, instead of the opposite relationship that we focused on. We found that GPs, who are more exhausted and dissatisfied with the available time, have longer consultations with more communication utterances. GPs with more feelings of exhaustion or depersonalisation discuss more psychosocial topics. Possibly, these GPs have negative feelings *because of *the intensity of their patient contacts. It is plausible to think that their more intense patient contacts actually cause GPs' negative feelings, instead of the opposite.

Furthermore, it is known that people who are most at risk of burnout, are impassioned people who are working hard. To demonstrate this by a quotation: 'In order to burn out, one has to be first 'on fire' [44]. GPs in our sample are the GPs who may have symptoms of burnout, but not so severe that they cannot work. The GPs in our study, showing high levels on some of the burnout scales, are possibly the hardworking GPs who are still 'on fire'; they invest a lot in their patients but are most at risk of burnout.

### Methodological considerations

The number of GPs with high levels of burnout is under-represented compared to GPs without burnout symptoms. This is especially true for the number of GPs with high levels of exhaustion, the core component of burnout. The mean scores on the exhaustion components of burnout are low compared to other studies reported in the manuals of the UBOS and MBI [12,37]. GPs in our sample suffer less often from feelings of burnout compared to other primary care workers. Of course, the most serious cases suffering from burnout are sick and off work and so did not have the chance to participate in our study.

In order to be sure that we identified GPs with low versus high scores on subscales of burnout, we did not analyse the continuous scores, but we used external norms for Dutch primary care providers to classify the GPs into low and high levels of the burnout components. The group of dissatisfied GPs is classified according to the meaning of the scale points, instead of in terms of a percentage.

Secondly, the causes and effects of the relationships studied are unclear, due to the cross-sectional design of the study. It remains unclear if the GP's communication and consulting style is affected by their feelings of burnout or dissatisfaction with the available time, or if the GP's consulting style influences the presence of symptoms of burnout or dissatisfaction. Although the results of this study make the latter explanation most plausible, the answer to these questions is that presumably both perspectives are partly right. In studying GP-patient interaction, it is plausible to think that GPs and patients influence each other in an iterative and responsive process [45], and in studying just one single relationship, these responsive reactions would be ignored.

Finally, differences in communication and other aspects of the consultation between GPs with low and high burnout and job satisfaction levels are presented after adjustment for GPs' and patients' age and sex. Significant differences might therefore be attributed to GPs' burnout or satisfaction, and not to the age or sex of the GP or the practice population. But it is useful to realise that combinations between GPs' or patients' age and sex and their levels of burnout or job satisfaction exist in 'real life'. These combinations might increase the differences between GPs with low and high burnout or job satisfaction.

## Conclusion

Firstly, we can conclude that GPs' feelings of burnout or dissatisfaction with their available time do not obstruct their diagnosis and awareness of patients' psychological problems. On the contrary, consultations from GPs with high levels of exhaustion or depersonalisation, and from GPs who are dissatisfied with their available time, can be favourable from the perspective of the patient with mental health problems. They provide longer consultations or talk more about psychosocial issues. As these GPs provide more opportunities to discuss mental health problems, this increases the chance that appropriate care will be found for patients with mental health problems. On the other hand, these GPs are themselves more likely to retire, or risk burnout, because of their dissatisfaction. Therefore, these GPs may benefit from training or personal coaching to decrease the chance that the process of burnout will get out of hand.

Secondly, GPs with feelings of incompetence create fewer conditions for patients with mental health problems to talk about their problems, by showing less affective communication, eye contact and patient-centeredness in their consultations. Attention to these aspects of communication in the training or personal coaching of medical students or practicing GPs may improve care for patients with mental health problems.

## Abbreviations

GP: General practitioner; DNSGP-2: Second Dutch National Survey of General Practice; MBI: Maslach Burnout Inventory; UBOS: Utrecht Burnout Scale; RIAS: Roter Interaction Analysis System

## Competing interests

The authors declare that they have no competing interests.

## Authors' contributions

EZ advanced the first conception of the study, performed the statistical analyses and drafted the manuscript. PV and DB participated in the conception of the study and assisted with the statistical analyses. KM and JB contributed especially to the interpretation and discussion of results. All authors read and approved the manuscript and gave final approval to the version to be published.

## Pre-publication history

The pre-publication history for this paper can be accessed here:


